# Societal Readiness Thinking Process 2.0: Incorporating Epistemic Reflexivity for Responsible Innovation

**DOI:** 10.1007/s11948-025-00568-7

**Published:** 2025-11-26

**Authors:** Robert Braun, Michael J. Bernstein, Arnab Chakraborty, Johannes Starkbaum, Florian Winkler

**Affiliations:** 1 Social and Sustainable Transformation Research Group, Institute for Advanced Studies - Institut für Höhere Studien, Josefstädter Straße 39, Vienna, 1080 Austria; 2 Center for Innovation Systems and Policy, AIT Austrian Institute of Technology, GmbH Gieffengasse 4, Vienna, 1210 Austria

**Keywords:** Societal readiness, RRI, AIRR, Science and technology studies, Epistemic reflexivity, Ethnomethodology

## Abstract

Frameworks for ascertaining the societal dimensions of research and innovation (R&I), such as the Societal Readiness Thinking Tool (SRTT), have supported reflection on ethics and responsibility but often risk reducing reflexivity to procedural checklists or impact assessments. This paper develops an enhanced version, the reflexive SRTT 2.0 process, by incorporating concepts of epistemic reflexivity and ethnomethodological sensitivity. We introduce the concept of *reflexive societal readiness*, which understands readiness as a situated, ongoing accomplishment shaped by both local practices and institutional “relations of ruling.” Drawing on ethnomethodological observations, reflexive questionnaires, and an initial workshop in the Horizon Europe project AGRO4AGRI, we examined how researchers engaged with reflexivity in practice. Our findings reveal three recurring patterns: reflexivity was often deflected through reliance on methodological safeguards, outsourced to societal impact experts or stakeholders, and substituted with compliance to regulatory frameworks or dominant imaginaries of sustainability and competitiveness. These practices uphold internal project orders and limit the potential for interdisciplinary learning and critical engagement. To address these obstacles, SRTT 2.0 proposes a reflexive process combining (a) observation of situated practices, (b) reflexive questioning that foregrounds individual positionalities, and (c) workshops that foster collaborative and institutional learning. This design enables researchers to critically interrogate their assumptions, engage more meaningfully with inclusion, and question the sociotechnical imaginaries shaping their work. We argue that embedding such reflexive processes into project lifecycles can extend and strengthen Responsible Research and Innovation (RRI) frameworks by cultivating collaborative, empathetic, and institutional learning. While challenges remain, SRTT 2.0 offers a transferable pathway for fostering more reflexive and responsible innovation practices.

## Introduction

Scientists and innovators have consistently focused on ensuring that their technologies perform intended functions; are taken up by societal actors; and markets respond favorably (Hjort & Brem, 2016; Mankins, 1995; Paun, 2012). In the past fifteen years, an increasing emphasis has been placed on incorporating broader societal and ethical dimensions of responsibility into research and innovation practices (Stilgoe et al., 2013; Von Schomberg, 2013). Most recently, these efforts have been combined by the European Commission under the banner of societal readiness.

According to its research funding Strategic Plan, the European Commission stresses that societal readiness, “Implies an interdisciplinary approach to projects, involving greater sensitivity and consideration about whether research and innovation” matches societal needs (EC, 2024, p. 39). The strategic document emphasizes, “[i]n addition to technological solutions, it is essential to address human, social (including the gender dimension), and societal readiness aspects for maximising societal, environmental, climate and economic benefits” (EC, 2024, p. 107). Operationalizing this statement, the European Commission has recently launched a “Societal Readiness Pilot” programme within Cluster 5 of its funding instrument Horizon Europe. Through an allocation of about EUR 80 million spread over 18 projects in a handful of topics on climate, energy, and mobility, the Societal Readiness Pilot initiative seeks to reorient research and innovation (R&I) practices by explicitly tasking project teams with identifying, reflecting on and responding to societal needs and concerns through all stages of work.

Integrating such concerns into research and innovation (R&I) involves consistent attention to process, as issues of responsibility necessarily change as projects progress (Owen et al., 2021). However, addressing the benefits and potential harms and ethical concerns of new technologies, while also actively anticipating, engaging and ensuring the responsiveness of innovations to societal needs and concerns remains challenging at institutional and individual researcher levels (Novitzky et al., 2020). Researchers studying Responsible Research and Innovation (RRI) sometimes critique the European Commission’s approach of six “keys” – ethics, open access, gender equality, science education, public engagement, and governance – as analytically weak, trading off conceptual coherence with seemingly politically viable, measurable concepts (Owen & Pansera, 2019). The procedural Responsible Innovation (RI) – a more bottom-up approach popular in particular in research contexts of the UK, Norway, the Netherlands, and science and technology studies communities – offers a possible resolution to this dichotomy of analytical rigor and pragmatic viability. RI emphasizes four process dimensions—anticipation, inclusion, reflexivity, and responsiveness (AIRR)—as guiding principles. This shift reframes innovation governance from narrow compliance with EU policy agendas toward a more systemic, open, and transformative practice of collectively stewarding science and innovation toward socially desirable futures (Griessler et al., 2022). The current EC Societal Readiness Pilot recognizes this advantage of AIRR and has integrated the procedural dimension into guidelines for projects in the pilot.

The procedural focus of Societal Readiness challenges the “lyseology” of R&I and its uptake (Braun, 2024). Lyseology, a version of agnotology (Proctor, 2008), constructs a present-day (social) challenge and posits that a (socially) challenge-free future depends on yet-to-be-developed engineered artifacts leveraging science. This argument is then used to persuade policymakers and the public to address said social challenge through technological innovation. Consequently, the lyseology then asserts that society must necessarily mold itself and openly accept said technological innovations to realize (socially) challenge-free futures. A procedural approach to Societal Readiness, focused on centering societal needs and concerns, holds that technologies must be designed and tailored to society.

Notions of “readiness” have been developed and formalized in a range of taxonomies to categorize and review technology innovation. The Technology Readiness Level (TRL), established in the 1980s, quantifies a technology’s maturity, providing an assessment framework for technical and economic costs, prospective value, associated risks (Mankins, 1995) on the path to societal uptake and commercialization. Other taxonomies, such as the Demand Readiness Level (DRL) (Paun, 2012) and Market Readiness Level (MRL) (Hjort & Brem, 2016), aim to gauge societal demand for technology which, within the lyseological framing, assumes a collective societal readiness to apply it.

In an attempt to move beyond lyseology and scrutinize whether innovations actually adequately address broader, long-term societal concerns, the concept of Societal Readiness (SR) has been introduced (Bernstein et al., 2022; Büscher et al., 2023). Büscher et al. (2023) have developed a Societal Readiness Assessment toolkit, originating in a decarbonization project, grounded in Jasanoff’s (2007) concept of “technologies of humility” and Haraway’s (2008) “response-ability.” The toolkit initiates a process that helps in addressing vulnerabilities, shaping and distributing risks, and fostering collaborative learning. It generates recommendations that encourage a deeper exploration of challenges, opportunities, and unexpected consequences, considering diverse perspectives (Büscher et al., 2023, p. 5140).

Grounded in the AIRR principles (anticipation, inclusion, responsiveness, and reflexivity) (EC, 2014; Mejlgaard et al., 2018), the Societal Readiness Thinking Tool (SRTT) [https://thinkingtool.eu/], developed in the Horizon 2020 project NewHoRRIzon [https://newhorrizon.eu/], is suggested to serve as a practical resource for scientists and engineers to integrate societal and ethical dimensions into their R&I (Bernstein et al., 2022, p. 6). The tool was developed out of a participatory evaluation and change oriented action research process to reflect on the deficiencies of integrating RRI keys into the European R&I Arena through its research funding programmes (Novitzky et al., 2020). The emphasis on “thinking” reflects an iterative activity across all phases of respective projects. The SRTT is designed to “spark thinking” at any project stage, encouraging users to “think across” societal issues, “think through” responses to these issues, and “think with” colleagues and stakeholders to modify practices accordingly.

Our paper presents the case of an EU agricultural innovation project (AGRO4AGRI), wherein the concept of societal readiness and a modified version of the SRTT is developed and implemented. We extend the concept and the tool to integrate a reflexive more-than-human approach to R&I processes (Braun, 2024). This is warranted as the lyseologist perspective has demonstrated considerable resilience against theoretical and political efforts to reconsider technological progress, power dynamics, and responsibility within R&I. Concepts such as “sustainability,” “corporate social responsibility” (CSR), and even some critical STS approaches towards responsible/ethical interventions, meant to promote greater reflexivity about the consequences of technological innovations, have often been absorbed into the dominant corporate power structures (Ehrnström-Fuentes & Böhm, 2022). This absorption supports the push towards lyseologist approaches, rather than offering genuinely transformative socio-political pathways centering broader societal needs and concerns.

Our ambition is informed by a turn towards performance, practice and an etnhomethodological sensitivity (Crawley et al., 2021) that sees research and innovation as doing: indexical and reflexive practices that are also inseparable from power/knowledge operations. Local, accountable practices temporarily stabilize the technoscientific order while drawing upon “extralocal relations of ruling” (Smith, 2001). Agency and order are co-constitutive, emerging in the dynamic interplay between situated coordination and action and the wider formations of power/knowledge that authorize, constrain, and make possible what can be enacted. Our inquiry and offering engages with this interplay.

Societal Readiness Thinking 2.0 – a process rather than a one-off tool, we propose, aims to address the impacts of and build researchers’ awareness towards power/knowledge structures, by critically engaging with the research process and its institutional setup (the “external relations of ruling”) as well as by integrating reflexivity about the constraints and potentials of this interplay throughout R&I processes.

### A Critique of Societal Readiness 1.0

Science and Technology Studies (STS) explore the interactions between scientific knowledge, technological innovation, and society. STS is concerned with why and how certain forms of knowledge and technology emerge (Nehring, 2021) and how technologies embody and replicate the values which shape them (Gugganig et al., 2023). As such, STS is sensitive to dynamics of power and the roles of various stakeholders involved in the co-production of science, technology and society (Braun & Starkbaum, 2022; Felt, 2016). Such a perspective is reflected in concepts like RRI and RI, but only to some extent.

RRI and RI as well, as the societal readiness concept to date, reflect a binary of research / innovation and society according to which R&I artefacts and knowledges are inserted into society conceived of as some kind of separate entity. As described by Bernstein et al. (2022), the SR Thinking Tool 1.0 pairs and situates the AIRR principles with the RRI “keys” in a way that invites greater nuance and detailing of social perspectives without necessarily challenging such a binary assumption. Consequently, as Braun (2024) argues, in its present form, the SRTT addresses R&I mainly as an unproblematic, ethically contained technological and economic issue. It thus does not radically challenge the lyseology of R&I. This traditional innovation and economic framing severely limits the SRTT’s capacity to radically challenge researchers’ and practitioners’ awareness of ways research and innovation shapes worlds and is embedded in and shaped by structures of power (Smith, 1999; Garfinkel et al., 1981; Latour, 1988, 2004; Mol, 1999).

Moreover, the concept of societal readiness, as implemented in the SRTT, is based on a somewhat pre-Kuhnian approach to the sociology of science as if it were a linear evolution towards (technoscientific) progress (cf. Kuhn, 1962). Also, it reflects a pre-critical theory approach to “the social” reified as something fix and object-like in relation to the readiness in general (cf. Adorno & Horkheimer, 1992). The “social” refers to the effects of the lyseological proposal embedded in technology and not the genesis of the technology that is being innovated (Pinch & Bijker, 1984). Put otherwise, the contribution of the SRTT seems to lie in addressing questions related to potential societal impacts as opposed to a detailed reflection on the socio-political construct that is the research process complete with its institutional embeddedness, as well as the presumptions and ideals of the researchers themselves (Woolgar, 1985, pp. 558–559).

Rather than conceiving of research as an abstract, linear progression culminating in societal effects, it is more accurate to understand it as an ongoing accomplishment: a form of doing and performance constituted in and through situated practices. From this perspective, “readiness” is not a measurable stage that technologies or projects pass through on their way to impact, but something continually achieved and displayed through performances, demonstrations, and inscriptions that must be made to count as credible in particular contexts. While the SRTT 1.0 acknowledges this reality, in tactically adopting the lyseology of R&I and seeking to be used as part of contemporary project processes, it has no capacity to break out of this false linear frame. Dorothy Smith’s (1999) insight into the articulation between local practices—such as what unfolds at the laboratory bench—and “extralocal relations of ruling” highlights how questions of readiness are never merely technical matters, but are deeply entangled with institutional, bureaucratic, and policy frameworks. Research practices acquire their meaning and legitimacy by aligning local accomplishments with these wider structures of governance and evaluation, which in turn shape what can be recognized as “ready.” Garfinkel’s (1967) notion of the *texture of relevances* further sharpens this view: a state of readiness may never be stipulated in advance but is instead accomplished contextually within shifting horizons of relevance, depending on the purposes at hand, the audiences addressed, and the accountability relations invoked. Against this backdrop, the SRTT 1.0 as a tool to add textured detail of the social dimensions of technical precisely misses this larger contextual, reflexive accountability through which readiness is enacted.

A reimagined *Societal Readiness 2.0* approach must move beyond linear scales, impact metrics, and questions of responsibility within the frame of lyseology to instead foreground the situated, performative, and reflexive nature of research practices. Such an approach would begin from the recognition that readiness is a locally accomplished performance and not an abstract ideal. It is continually negotiated, exchanged and mutually co-constituted by researchers themselves through demonstrations, inscriptions, and alignments with institutional and policy expectations. It would therefore require mechanisms that attend to the interplay between local practices and extralocal relations of ruling, acknowledging how the laboratory bench and the policy arena are co-implicated in producing what counts as “ready.” In line with Garfinkel’s emphasis on the texture of relevances, Societal Readiness 2.0 needs also to be sensitive to the shifting horizons of accountability that researchers navigate, where different audiences (funders, regulators, stakeholders, publics) make different relevances salient. Practically, this implies designing readiness assessments as reflexive processes rather than static checklists or one-off questionnaires: processes that invite researchers to critically interrogate how their own practices, imaginaries, and institutional contexts shape the enactment of readiness. Instead of treating the social as an external domain of impacts, readiness would thus be redefined as a socio-material and performative achievement, co-constituted through the ongoing work of making research accountable, credible, and legitimate across multiple sites of practice.

### Reflexivity

Reflecting on ways of knowing involves using “thinking tools” that focus on relationships as the core of human actions. This approach highlights that agency (individual performances and interactions), and “ruling relations” (societal rules) are intertwined, moving beyond seeing them as separate or fixed (Smith, 1999, 2001). Relational analysis suggests that the (social) world consists of relations (Smith 2005) and interactions (Goffman 1983) rather than isolated things. If researchers don’t question the established modes (the “ruling relations”) and the perspectives (the seemingly fixed “texture of relevances”) they use to understand and address social phenomena, the knowledge they produce will likely blindly reinforce the institutional processes that organize and coordinate everyday (research) activities, linking their local practices to wider systems of power and the imaginaries power elicits.

To bring such a relational perspective, a radical reflexivity, “Enjoins the analyst to displace the discourse and practices that ground and constitute their endeavors to explore the very work of grounding and constituting. Intrinsic to radical reflexivity is an ‘unsettling,’ i.e. an insecurity regarding the basic assumptions, discourse and practices used in describing reality” (Pollner, 1991, p. 370). This entails challenging the very basis of a traditionalist, western mode of doing science, as it has been done in various ways within the social sciences and humanities of the past hundred years (e.g., Husserl, 1970; Derrida, 2016; Garfinkel, 1991; Butler, 1999; Escobar, 2020a, b). Consequently, we ground our approach in Bourdieu’s concept of epistemic reflexivity: the scientific objectivation of the subject of objectivation (Bourdieu, 1978). Objectivation involves scrutinizing the researcher through turning the tools of analysis back onto the researcher themselves. With this approach, researchers can achieve reflexive research through a systematic exploration of social scientific knowledge claims by scrutinizing their own self, cultural practices, biases and unthought categories of thought. This concept refers to the implicit, taken-for-granted frameworks and assumptions shaping how people – often unconsciously – perceive and interpret the world.

So grounded, reflexivity addresses individual researchers’ positions as well as the visions and communal affiliations or artefacts that constitute the edifice of “Big Science.” The “turn to reflexivity” has long shaped STS, particularly in the sociology of scientific knowledge (SSK), where it has been used to interrogate both science and its study (Ashmore, 2015). Ashmore (1989), Mulkay (1985), and Woolgar (1988) treated reflexivity as an opportunity; Latour (1988) cautioned against “meta-reflexive” strategies and advocated instead for “infra-reflexive” attention to ordinary practices. The 1990s brought critiques of reflexivism’s limits (Pinch, 1993; Winner, 1993), while Lynch (2000) systematized reflexivity into six forms, favoring the ethnomethodological as a mundane feature of social life. Through reflexivity, individuals can begin to recognize and question these hidden influences, potentially leading to more critical and transformative thought. It involves two steps: distancing from the research situation itself and stepping back from the act of observing. This approach requires deep self-reflection beyond just noting basic personal details like gender, nationality, or profession. Bourdieu’s approach avoids self-indulgence and instead aims to increase awareness of the often-unnoticed values and assumptions that researchers bring to their work (Guttormsen & Moore, 2023, p. 539).

To operationalize this form of reflexivity, we evoke the idea of co-creative learning or collaborative knowledge-making. Early action researchers have argued that by blurring the positions of system insiders and researchers, “co-generative learning” spaces may be created (Elden & Levin, 1991). Co-generative learning in action research contrasts with top-down knowledge production by involving researchers and participants in joint cycles of reflection and action. Rather than imposing solutions, it integrates multiple perspectives to create context-specific understandings and practices that are socially robust and practically relevant, with awareness of the sometimes-diverging goals on either side of scientific and broader social agendas.

### Reflexive Learning in Agricultural Innovation

Such an STS-inspired reflexive approach is currently applied in the Horizon Europe funded agricultural project AGRO4AGRI. The project seeks to deliver solutions for plant nutrition and protection consisting of nano and biobased controlled delivery fertilisers and plant biostimulants, and target-specific biopesticides based on RNAi technology, each suggested for enhanced agrochemicals use efficiency. In the lyseology of R&I, it is an early-stage innovation people aspire to move from technology development to product design and exploitation. As such, it provides an opportunity to integrate reflexivity at all crucial stages of development till mid-2028. In this paper, we document our efforts in the initial stages of the project, which includes identification of materials and designing environmental assessment frameworks.

Innovation has always been at the center of agriculture, from incremental innovation, such as trial-and-error breeding to technology-driven innovation such as gene editing (Gremmen et al., 2019). It has been pointed out that political economic factors globally (Kumbamu, 2020) and in European agriculture (Ody & Shattuck, 2023) have led to the prominence of a techno-solutionist/lyseologist trajectory, which incentivizes developments in specific forms of agro-chemicals. This trajectory emerged from WWII war industry and has fueled the human degradation of ecosystems around the planet (Hayes & Hansen, 2017). Innovation in agriculture is based on a much older modernist project and, inter alia, is being increasingly challenged by indigenous knowledge systems and movements (McMichael & Schneider, 2011; Pouchepadass, 1995). Furthermore, there are unevenly distributed harms and responsibilities resulting from a modernist agriculture (Cusworth et al., 2023), which call even more for a reflexive perspective.

Earlier participatory action research in agriculture was designed to ensure effective adoption and scaling of existing innovations, while bringing incremental changes within the innovation system (Foote Whyte, 1991). The more recent attempts at agricultural technology development from the margins have been designed with a more reflexive approach by including institutional learning systems within the innovation process, and allowing for reflection on the positionality of the researchers as well as the farmers and other practitioners involved (Prasad et al. C, 2025; Snapp et al., 2023). But these remain at the margin, within niche projects. The Big Science in the agricultural sector is still inflicted by “chronopolitics,” i.e. the temporal order through projectification of research, and how academic careers are strictly timed drive academic work and knowledge production (Felt, 2017b). Even the advance of RRI approaches is circumscribed by R&I governance framings of acceptable research, valuations of certain activities and outputs over others, and artificially partitions of work into administrative logics (Smith et al., 2023). Thus, the given epistemologies and ontologies in academia and research may prevent ethical reflection and “potentially doing things different if needed” (Prutzer et al., 2023, p. 5).

The AGRO4AGRI consortium, formed in response to a call issued by the European Commission in its Horizon Europe funding stream, represents an example of Big Science promoting industrial agricultural input development. As the call also prescribed social science and humanities (SSH) engagement in the project, our intervention aims to bring a reflexive approach to designing agricultural technologies by integrating reflexivity, iterative co-generative learning cycles within the project, while developing a new process-based SRTT.

### Developing the SRTT 2.0: Reflexive Societal Readiness Thinking

Our proposed further development of the SRTT 1.0 retains the idea of, “A practical resource for scientists and engineers who wish to integrate broader societal and ethical dimensions of responsibility into their practices” (Bernstein et al., 2022, p. 6). We also retain the important proposition of ‘thinking,’ “as a central, iterative activity unfolding across phases of research and innovation projects” (ibid.). We diverge by engaging more deeply with Bordieuan epistemic reflexivity as well as a Smithian ethnomethodological sensitivity towards the interplay of local performance and extralocal relations of ruling. Bringing forward SRTT 2.0, we suggest it is not enough to “spark thinking” at stages of a project lifecycle as if the project was a “thing” in a world independent of a researcher, their knowledge, bias or social context. We suggest it is not enough to “think across” a range of societal issues separated from a researcher and their imaginaries; nor enough to “think through” responses without reflecting on the hidden values and assumptions that researchers bring to their work. Finally, we suggest it is not sufficient to “think with” colleagues and stakeholders on how to respond with modifications in practice without discursively questioning the assumptions, biases and the texture of relevances – the shifting, situated web of concerns, priorities, and meanings through which “members” make sense of and organize everyday activities – of such groups and the people involved.

When redesigning the tool and its questions to the context of agricultural innovation, we embedded it in a process involving field research and reflexive workshops. Our ambition is to offer a practical and transferable reflexive process by which researchers in a specific R&I project can critically engage with their own positionality, epistemic biases and cultural/political contexts, as well as the ruling relation, and thereby increase awareness of often-unnoticed values and assumptions that researchers bring to their work.

The main ambition of the thinking tool/process 2.0 is to help researchers and innovators to “think across/through and with” their local performances at the laboratory bench/research venue. The charge of SRTT 2.0 is to do this with acknowledgement of the interplay of extralocal relations of ruling – technological imaginaries, policies, funding and project expectations, etc. The idea here is to avoid having participants legitimate their activities by the lyseology of the paradigmatic scientific community. In this context, “thinking” requires a researcher to access a self beyond the procedural, lyseological expert. They must access a responsible researcher/innovator who would think *across* social worlds with fellow researchers and the wider social field they interact with. They must access an interactive researcher/innovator to think *through* potential ethical implications or impacts. They must access a performatively engaged researcher/innovator who shares concerns and learns *with* colleagues who may have – and also work to overcome – similar biases and hidden assumptions. Our focus is not on the individual and their “ideas” but rather, in line with our ethnomethodological sensitivity, on their actions as reflexively surfaced, indexically tied to context, and rendered accountable and intelligible to other researchers.

The process involved three steps. First, participants were involved in ethnomethodological observations across the project life cycle to develop a knowledge base on the phenomenal field (Garfinkel, 2021, Eisenmann et al. 2021). Second, participants involved were invited to implement the SRTT 2.0 process and “think across” and “through” actions and interactions in the project through an open questionnaire. Finally, participants were involved in a series of reflexive workshops with researchers and innovators to “think with” colleagues beyond the lyseology and project-life constraints.

### Ethnomethodological Observations

Ethnomethodology is the study of ethnomethods, i.e., “the formal properties of common-sense activities as a practical organizational accomplishment” (Garfinkel, 1960) or the observation of the work “that makes up the produced witnessability” (Wiley, 2019, p. 168) of ordinary social facts of life. An ethnomethodological sensitivity (Crawley et al., 2021) emphasizes that reality is not a fixed given but an accountable, discursive-material accomplishment of (social) order. Garfinkel’s concept of “member” refers not merely to an individual (in our case the researcher) but to a socially competent actor who reproduces intelligibility through shared procedures of sense-making. Membership is thus practical and situated: knowing what counts as relevant, factual, or orderly and acting so that others can recognize it as appropriate. Dorothy Smith’s (1999) notion of “relations of ruling,” referred to previously, highlights how such local practices are shaped by extralocal institutional arrangements. Here, ethnomethodology connects to STS: Garfinkel’s studies of the “discovering sciences” showed that facts emerge through accountable practices of order-making; Lynch demonstrated how laboratory instruments and notes are embedded in local methods; Knorr-Cetina (1981) traced distinctive epistemic cultures; and Latour and Woolgar (1979) revealed how inscriptions stabilize claims. Together, these studies underscore that science and technology are constituted through situated, historically specific practices of seeing, reasoning, and recording.

Ethnomethodologists observe peoples’ ways of accounting to themselves for certain matters. Performances are studied to see how reflexive and accountable sense-making occurs. Studying ethnomethods (accountable and reflexive performances) seeks to understand the modes by which individuals construct, negotiate, and agree upon reality. Such an approach focuses on members’ methods for making visible, demonstrable, and accountable the particular settings’ features, e.g., how they recognize, describe, and make accountable, for themselves and each other, such matters as the research object, the research question, research subjects, stakeholders, regulations, imaginaries, procedural limitations and all other features that are deemed to be important for the research project at hand.

In the SRTT 2.0., an ethnomethodologically informed observation is the first step as well as an ongoing process involving the ethnography of the ethnomethods of researchers and innovators. As practical resource in a project, we suggest that project documents and the project kick-off meeting serve as an initial place of observation of the “ethnos” – group of researchers and innovators involved in the project (aka “members”). It is the kick-off where the whole of the project is discussed, and different tasks explained. Ethnomethodological research includes field notes of “discoveries” with regard to a set of members’ practices, e.g., what and how categorization devices are used, how things are ‘counted’ (traced, located) or not counted; and how persons, things, beings are classified and unclassified, collected and separated. The field notes and the analytic discussions serve as input for designing the Reflexive Societal Readiness Thinking process (SRTT2.0).

### Findings from Ethnomethodological Observations

The SRTT 2.0 was developed and applied as part of an EU agricultural project that began in 2024. The kick-off was situated in the project coordinators’ headquarters, a scientific research laboratory with glass walls behind which scientists in white robes worked at their benches. “Sustainability” was present in conversations and the surrounding space. Instead of conference bags, participants got a pencil that had seeds in it to plant; the name tags on the lanyard were made out of carton which one can rip apart and put into soil to let some plants grow. The UN Sustainable Development Goals (SDGS) were painted on the wall of the conference room. All of these are textual extralocal relations of ruling that become operationalized in the doings of the researchers at their laboratory bench or discussion venues such as the kick-off. During the project overview, participants were introduced to the framing of pesticides: they have a huge runoff and the way they currently work, they produce economic loss and pose health threats to humans. The project was positioned to counteract these economic and human wellbeing threats.

Another declared main objective is to eliminate, or to “inhibit reproduction of nematodes,” which typically described using passive and elusive language like “inducing lethality.” One presenter talked about their technology (nematicide and microsencapsulation) as being risky and a difficult one, but “that is why there is funding by the European Commission.” That R&I inherently contribute to the public good was a shared assumption in all meetings. Legitimization for the project was also derived from its broader ambitions, such as supporting the SDGs or addressing food needs and hunger.

As in most EU R&I projects, work is structured along work packages and tasks. This “projectification” encourages a focus on micro-management – addressing (technical) problem-solving and smaller socio-ethical questions step-by-step rather than addressing broader structural aspects of agriculture. Regulatory frameworks, such as the Safe and Sustainable by Design (SSbD) principles, which address the potential toxicity of materials, are applied to address socio-ethical aspects.

In several project meetings we observed a set of mundane practices such as:

- ”*Dealmeaking*” – commonsensical negotiations take place between bureaucratic and scientific accomplishments in order to “get things done” and “meet deadlines”.
- “*Deliverification*” – operationalizing projectification in a way that whenever problems or questions arise, scientific uncertainties are settled bureaucratically in order to meet deliverable deadlines and formats.
- “*Suspending belief*” – outside reality, general societal concerns or conditions are bracketed and only the reality of the project and its predefined research and innovation ambitions guide the work of the researchers.

These practices became visible in a meeting when the role of non-target organisms was discussed. The following was stated: “We have to make sure that non-target organism are not affected”; to which another person answered: “Of course, it will be modelled and predicted.” Someone intervened: “We can’t predict with 100% certainty, but we will still predict.” So, the question arose: “Can we test impact on non-targeted nematodes?”. “No”, –another person said, “not in this project, there are several hundreds of them, we can’t test, we have no time.”. “So how can we do it?” – asks someone. “We will model and predict.”

The vignette of the SRTT 2.0 kick-off meeting demonstrates how local performances—scientists at their benches, presentations, exchanges over terminology, even the symbolic use of “green” objects—are saturated by extralocal relations of ruling such as the UN SDGs, Safe and Sustainable by Design principles, and the economic agendas of the European Commission. These frameworks are not abstract backdrops but are made operational in mundane practices of “dealmaking,” “deliverification,” and “suspending belief.” What counts as relevant or accountable in the moment (e.g., predicting effects on non-target organisms rather than testing them) emerges in this alignment between local accomplishment and broader institutional logics. Individual actors must navigate uncertainties, making their statements accountable within project deadlines and formats, while collectively the project participants reproduce the legitimacy of the endeavor by aligning it with extralocal discourses and regulatory expectations. Reflexivity, even when applied, here does not take the form of deep societal self-questioning but of pragmatic adjustments that stabilize the project’s trajectory. Thus, “readiness” at this stage is about sustaining a workable order at the intersection of scientific practice, bureaucratic management, and political aspiration.

### Implementing Societal Readiness Thinking (SRTT) 2.0

#### Epistemic Positions and Reflexivity

The next step in the SRTT 2.0 process, an online questionnaire addressed to every member of the consortium, emphasizes situated knowledge and engagement within the local and the interplay with the extralocal, and is adapted to the specific innovation case. Questions are designed to connect with the four dimensions anticipation, reflexivity, inclusion and responsiveness (AIRR) of Responsible Innovation (Owen & Pansera, 2019; Stilgoe et al., 2013).

The questionnaire is an online space that provides several (closed and open-ended) questions for researchers and innovators. Other than in a survey, the main ambition is not to collect opinions or normatively “proper” answers but to provide a space for reflexivity on one’s own actions. Responses serve as a knowledge base for the subsequent workshop series. This ambition is explained at the outset in clear language along with informed consent obtained from the researchers. Questions are grounded in the AIRR principles and intend to provide a reflexive space for engaging with power structures and established ways of thinking. Questions like “What do you personally think: Who benefits most from expected impacts of *your work”* address each person individually and put their actions into focus. What demarcates the questions posed in the SRTT 2.0 from the “thinking” questions posed in SRTT 1.0 is that they are informed by an ethnomethodological sensitivity: focusing on the doings of researchers that are reflexively produced, indexical, and accountable making them intelligible to other researchers. Questions are also tailored specifically to the context of the concrete research project (in this case AGRO4AGRI).

Responses to the open-ended questions were coded by two researchers in sequence, building on each other’s inferences in a discursive process. After the final coding stage 16 broad codes were identified covering 223 quotes. Multiple reflexive discussions within the research team were held during and after the coding process that helped group these codes into themes discussed below. The reflexive discussions helped identify how the codes coincided with the AIRR principles and helped separate and cluster empirical evidence in the coding process (cf. V. Braun & Clarke, 2019).

### Key Findings

Throughout the analysis and its grounding in the AIRR principles, three thematic areas were identified.

#### Foreclosing Responsibility with Methodology

Here we look at how the respondents revealed their epistemic considerations about their research subject, and the project, in the process of anticipating research outcomes. The analysis also highlights how these considerations are at odds with the process of self-reflexivity.

Overall, most participants express confidence in the planned R&I activities and its impacts without actually having a shared vision or actually *doing* anticipation: “*The developments planned will be positive for the nature*” (Case 3). The assumptions are that the use of appropriate scientific methodologies will make the anticipation of the outcomes comprehensive and objective: *“We could estimate the impact of the product on the farmers performance better and which products might not be used because of the project. Others could estimate this impact on stakeholders….“* (Case 7). In this respect, performances like impact assessment, life-cycle assessment, measurement or optimization are often invoked as methodological safeguards for reducing potential negative outcomes that can be attributed to the technologies. These rely on the assumption that the negative impacts are measurable, and assessable “objectively” and individually. The assumption remains consistent, even when posing the possibilities of paradigm change or “green” transition. However, in some instances, the respondents indicated reasonable doubts about the scope of their methods: “*As in other research processes I can anticipate about specific aspect related to the topic. I synthesize*,* characterize and test some materials for a specific application. Any other aspect not related to the topic is difficult to be considered. The slow release of fertilizers as the actual way that fertilizers are applied have uncertainties about the overuse of grounds”* (Case 23).

Another common but less frequently stated epistemic pathway to anticipation is the use of a collaborative engagement approach. It extends the methodological issues to the use of more participatory methods where the research can be made more responsible, *“By keeping a record of stakeholders*,* societal groups*,* and others likely to be impacted by the project’s outputs*,* and by communicating with all consortium members to understand their views on the value propositions to these groups*,* we can gain clearer insights. Many of these impacts can also be quantified (in monetary*,* carbon*,* biodiversity*,* or health economics contexts) allowing for more informed consideration.”* (Case 4). However, in this approach reflexivity is outsourced to other parts of the team or stakeholders: *“I’d like to hear more from the societal impact experts.“* (Case 20).

Another relation for a few respondents was the regulatory regime. They used the adherence to regulations as a means of addressing societal responsibilities of their research. While some scientists see their work as creating new knowledge that can inform regulation, others consider adherence to regulations and standards as a form of responsible research principle, therefore a substitute to reflexivity.

#### Inclusion Without Participation

Most of the responses describe inclusion as a process of learning about perspectives of others, yet in a surprising number of instances, without the actual voice of these parties. Some respondents frame inclusion as an act of care by highlighting the importance of varied needs in the abstract, thus acknowledging the role of inclusion as more than a tool for avoiding the ethical pitfalls within the project and demonstrating empathy for the unknown end-users. Other respondents also acknowledged that the project’s impact can be spread across the ecological system: *“Any change*,* however small*,* will affect soil biology and soil interactions for better or worse*,* thus affecting everything from soil micro-organisms to micro-organisms found in the human body.”* (Case 17). Some even frame the entire project in non-anthropocentric terms, highlighting that *“the [project’s] scope is to interrupt by means of new modes of actions the parasite relationship between the nematode and the crop”* (Case 10). However, several narratives are accompanied by techno-solutionist assumptions that frame them as solvable problems, *“I account for various needs by evaluating effects on animals and the environment and adopting measures that address diverse needs and minimize harm”* (Case 11). In such cases, the researchers assume the position of accounting for inclusion, rather than finding ways to represent – concretely or abstractly or by proxy – different stakeholders in the process. Roles of different stakeholder groups are also pre-differentiated, without necessarily grounding in the voice of actual persons, as the following narrative on expected positive impacts demonstrates: *“Firstly*,* EU citizens*,* as food safety will be improved. Secondly*,* those companies that exploit the results through patents*,* as they will expand their ways of incomes.”* (Case 22).

#### Circumscribed Responsiveness

Overall, few strategies for adaptations within the institutional framework of the project were described. Rather, we observed more often the shared understanding that technological goals and solutions are required for overall socio-economic wellbeing of the majority of the population: *“I am happy to feel we are contributing to more sustainable solutions that will help preserving the environment while contributing to crops growth. This means helping the problem with food*,* contributing to health and contributing to mitigate climate change”* (Case 9). The ambition to contribute to a more sustainable way of agriculture has been repeatedly paired with economic considerations, as another narrative demonstrates: *“My assumptions about sustainability and competitiveness guide my work by shaping the focus of my research and decision. I prioritize approaches that balance sustainable practices with economic viability. At the same time*,* it is important to understand the need of agriculture and to explore innovative solutions to remain competitive globally. These assumptions influence how I evaluate technologies*,* strategies and policies”* (Case 11).

Institutional criticism usually focuses on few goals, such as communication and timeline issues. While, some institutional aspects were highlighted with overtly reflexive stances, project framing gets internalized as a limit on agency to respond to critique: *“The goal of the project should be focused on sustainability rather than profit*,*”* (Case 22) and *“I would like to stress that many of the ’critical‘ aspects that I may have voiced towards our project*,* are mostly related to the scope of the call for project application. It would not be possible to address the challenges that European agriculture faces currently and include more aspects of the challenges within the scope of the call“* (Case 12). In this way, participants sense of capability to respond to reflexive considerations is curtailed directly by the lyseology of the project form in which they are inscribed.

### Reflexive Workshops

A series of three annual reflexive workshops involving the consortium are planned during the AGRO4AGRO project. These will build on and continue the process initiated through the SRTT 2.0 process of ethnomethodological observation and reflexive questioning. These workshops will allow feedback to be given to the whole consortium and enable ideas to be co-created for aligning R&I activities with societal needs. The workshops aim to provide a space for researchers and innovators to critically engage with their own positionality, epistemic biases, and cultural and political contexts. Most importantly, they aim to raise awareness of the often-unnoticed values and assumptions that researchers bring to their work as well as the extralocal relations of ruling that inform and orient their doings. The main ambition is to encourage individual researchers to “think across, through and with” *their own* positionality, epistemic biases and cultural/political contexts in exchange, interaction, and relation with other members of the research team to engage in various forms of learning to actually do reflexivity.

At the time of writing this article, one three-hour workshop involving around 20 consortium members has been conducted. This workshop provided an opportunity for group members to reflect on their roles and practices as individual researchers and innovators, and to explore the possibility of learning together as a group and from others beyond it. Participants formed four small groups and were tasked with ideating about the food system in 2049, to learn about common and diverging visions that guide R&I. The groups developed various ambitions for the future involving lab-grown meat, improved fertilizer efficiency and modern weed management techniques.

In the final part of the workshop, participants were asked to consider ideas for learning in future R&I initiatives. Based on our earlier findings, these ideas were categorized into three types of learning: collaborative, empathetic and institutional. These addressed questions relating to working together, taking different perspectives and needs into account, and considering systemic and institutional aspects relevant to R&I and potential adaptations. The main ambition was to reflexively turn away from lyseology and engage productively with the ruling relations. Challenges identified included a dominant focus on technology and the European context across the project, which influence visions and ideas for aligning R&I. Several participants expressed a desire for more knowledge sharing within the consortium.

## Discussion

The ambition of developing SRTT 2.0 was to move beyond the limitations of existing readiness frameworks that treat societal aspects as external “impacts” or as checklists of compliance. Our findings show that while researchers and innovators engage actively with concepts such as sustainability, regulation, and inclusion, these are often mobilized in ways that deflect, outsource, or substitute more radical reflexivity. This points to a persistent tension: local practices of project work are saturated by extralocal relations of ruling—policies, funding logics, and institutional expectations—that stabilize technosolutionist trajectories and narrow the scope of reflexive engagement.

The ambition of the SRTT 2.0 reflexive process was to enable an interdisciplinary approach by inclusive participation throughout the project involving greater sensitivity and consideration of researchers to societal needs and to foster the potential of various forms of mutual learning. Our observations and analysis showed that various mundane epistemic practices were mobilized to produce scientific and project orderliness. We found that researcher’s adoption of local (“projectified”), occasioned (“sustainable”; “deal-making”), sequentially organized (“deliverified”) and embodied (“studying risks”) work limited reflexivity. Various extralocal relations of ruling (assumed economic and food-delivery benefits; SDGs, various imaginaries) also impinged upon their doings. Extralocal relations further limited their ability to reflexively engage with the interplay of their doings and its societal consequences as individuals and as members in a local research team. This, in turn impeded their ability to engage in various forms of mutual learning that might, ideally, be a core aspiration of multidisciplinary collaborations.

We propose the notion of *reflexive societal readiness* to capture an alternative orientation. Reflexive societal readiness is the ongoing, situated accomplishment of aligning research and innovation practices with societal needs through critical engagement with local practices and extralocal structures. It moves beyond linear readiness scales or external impact assessments by foregrounding epistemic reflexivity (scrutiny of researchers’ own assumptions, biases, and categories of thought) and ethnomethodological sensitivity (attention to how accountability and order are locally produced). Reflexive societal readiness thus emphasizes readiness not as a static stage, but as a performative process that combines individual reflexivity, collective learning, and critical engagement with dominant sociotechnical imaginaries.

In this perspective, readiness is not only about anticipating external impacts but about interrogating how scientific and innovation practices themselves co-produce societal futures. Our analysis highlights three domains where such reflexive readiness can be operationalized:

- Opening methodology towards anticipation – moving beyond reliance on methodological safeguards or regulatory compliance as substitutes for reflexivity, towards constructing anticipatory practices to better expose, critique, and alter the local and extralocal constraints.
- Radical participation– shifting from abstract or instrumental stakeholder inclusion towards practices of empathetic and relational participation that acknowledges ecological and non-human entanglements as part of innovation process.
- Re-bound responsiveness – recognizing how dominant imaginaries of sustainability, competitiveness, and food security rule relations of research trajectories, and cultivating spaces for co-generative learning that allow alternative imaginaries to surface.

Together, the above articulate SRTT 2.0 not as a checklist or survey, but as a reflexive process of turning against lyseology: a cycle that combines (a) ethnomethodological observation of project practices, (b) reflexive questioning directed at individual researchers’ positionalities, and (c) workshops that facilitate co-generative learning across the consortium. The methodological contribution of SRTT 2.0 therefore lies in embedding reflexivity into the temporal flow of a project, rather than treating it as an add-on exercise.

Our initial application in the AGRO4AGRI project demonstrates the challenges and the potential of this approach. While technosolutionist logics and projectified routines often constrained reflexivity, moments of critical questioning and desire for deeper collaboration emerged. This indicates that SRTT 2.0 can create openings for mutual learning even within the constraints of large-scale innovation projects. By articulating reflexive societal readiness, we thus contribute to the refinement of the SRTT to engage – rather than side-step – the lyseology of contemporary R&I systems. We suggest that reflexive processes foregrounding the individual researcher as a situated, interactive epistemic subject into project structures can strengthen the AIRR principles without losing sight of the collective and institutional dimensions of and a critical engagement with relations of ruling that dominate research.

The existing AIRR framework, while valuable, remains vulnerable to what Braun (2024) describes as lyseological tendencies of modern technoscience: the tendency to reduce responsibility to procedural compliance or to equate societal readiness with technological uptake. Our findings suggest that fostering reflexivity requires more than embedding AIRR principles; it requires cultivating co-generative learning strategies (Elden & Levin, 1991) that are context-sensitive and grounded in the epistemic cultures of specific technologies. Based on our analysis, we propose three interlinked strategies for managing reflexive learning in the project in response to the ethnomethdological challenges observed when introducing reflexivity in the SRTT 2.0:

- Opening methodology towards anticipation
  *Empirical Insight*: Reflexivity was frequently *deflected* by invoking the scientific method or regulatory assessment as sufficient safeguards.
    *Learning Strategy*: Encourage researchers to go beyond methodological assurance by asking how their anticipatory practices are themselves shaped by disciplinary assumptions, project logics, and institutional expectations. This requires creating safe spaces where researchers can reflect on uncertainties without pressure to resolve them instrumentally and propose or engage in alternative practices.
- Radical participation
  *Empirical Insight*: Inclusion was often *outsourced* to stakeholders or reduced to technocratic principles such as food safety or harm minimization, while care was *substituted* by commercial or institutional benefits.
    *Learning Strategy*: Reframe inclusion not as a procedural obligation but as a practice of empathetic engagement with diverse human and non-human actors. This involves cultivating attentiveness to ecological entanglements and fostering exercises that foreground relational responsibilities, rather than treating inclusion as an external box-ticking task.
- Re-bound responsiveness
- *Empirical insight*: Progressivist and developmentalist imaginaries of sustainability, competitiveness, and “green” growth were largely accepted uncritically, yielding power to what Latour (2010) calls *factishes*—socially fabricated visions that combine belief in technological progress with the authority of facts.
  *Learning Strategy*: Support researchers in identifying, questioning, and even reimagining the dominant visions that orient their work. This can be done by mapping imaginaries explicitly (e.g., through visioning exercises) and exploring alternative trajectories, thereby creating opportunities for institutional learning rather than simply reproducing the objectives of funding calls.

Taken together, these strategies extend the STS critique of limited reflexivity into practical pathways for managing learning within innovation projects. To operationalize them in AGRO4AGRI, we propose three measures:

- Broaden the reflexive questionnaire beyond the consortium to include external stakeholders, thereby bringing in alternative epistemic and ontological standpoints.
- Embedded visioning exercises in reflexive workshops, allowing participants to articulate and contest different imaginaries of agriculture and food futures.
- Institutionalize moments of collective critique within project governance (e.g., interim review sessions), ensuring that epistemic anticipation, inclusion as care, and engagement with imaginaries are not treated as add-ons but as integral to the project’s learning process.

In this way, reflexive societal readiness becomes more than a critical diagnosis: it is a managed process of collaborative, empathetic, and institutional learning that can resist the displacement of reflexivity into technosolutionist routines and open space for alternative futures. The point here is not simply to show how methodological framings can foreclose responsibility, but to demonstrate how they might also be mobilized to open responsibility. Such an orientation prepares the ground for more radically transformative interdisciplinary collaborations, where divergent epistemic and methodological commitments are not prematurely harmonized but held in productive tension. This matters because only in such conditions can genuinely new forms of knowledge and practice emerge.

At the same time, the argument carries significance for policy engagement. By offering constructively critical feedback to European Commission project officers and policymakers, researchers can contribute to shaping future agendas without abandoning institutional frameworks altogether. Rather than rejecting such structures, the emphasis can shift to a focus on influencing their trajectories, carving out spaces for more reflexive forms of research to gain legitimacy within the terms of policy discourse.

Finally, the discussion highlights the room for manoeuvre within existing project architectures. With determination and communicative eloquence, researchers may find ways to meet contractual obligations while still cultivating more reflexive and responsible practices. In short, the value of the SRTT 2.0 lies in showing that methodology need not foreclose responsibility; it can, instead, be the very means by which responsibility is opened, enacted, and sustained in the pursuit of more just and generative research futures.

At the same time, we acknowledge the limits of our approach. Co-generative learning strategies may enable reflexivity within established epistemic cultures, but they do not by themselves guarantee a radical questioning of the deeper structures that sustain technosolutionist trajectories. The durability of dominant imaginaries and institutional incentives means that alternative ontologies often remain marginal. Whether SRTT 2.0 can strengthen reflexivity sufficiently to shift these dynamics remains an open question—one that our continued engagement with the AGRO4AGRI consortium will help to clarify.

In sum, we offer SRTT 2.0 as a reflexive extension of SRTT 1.0, one that integrates empirical sensitivity, theoretical critique, and practical modalities for learning. It also highlights the opportunities that exist within current project structures: with persistence and clear communication, researchers can meet contractual obligations while introducing more reflexive practices. Responsibility, in this sense, is not only about compliance but also about agency—working within the system to deliver high-quality results while also opening the possibility for more transformative forms of collaboration.

## Conclusion

Our empirical work in the AGRO4AGRI project revealed how reflexivity is often constrained in practice. We observed three recurring practices working to stabilize project routines and limit the potential for interdisciplinary learning and critical engagement of societal consequences: *deflecting* reflexivity with the shield of methodological choices; *projecting* reflexivity of stakeholders without substantial participation; and *curtailing* reflexivity as compliance with the hegemonic imaginaries of research and innovation. To counter these tendencies, we introduced the concept of reflexive societal readiness: a situated, ongoing practice foregrounding three learning domains—epistemic anticipation, inclusion as care, and engagement with sociotechnical imaginaries.

Cultivating reflexivity beyond the constraints of lyseology, the SRTT 2.0 process offers an approach combining (a) ethnomethodological observations of local practices, (b) reflexive questioning to foreground individual positionalities, and (c) co-generative workshops to foster collaborative and institutional learning. SRTT 2.0 aims to re-center the individual researcher as a situated, interactive epistemic agents, to open new possibilities for collaborative, empathetic, and institutionally aware learning within large-scale innovation projects. This, we argue, is an essential step into a radical reorienting toward just and sustainable futures that could be enabled, rather than foreclosed, by R&I.

## Data Availability

All relevant data has been included in the manuscript.
